# Analysis of the Influence of Different Diameters of De Laval Supersonic Nozzles on the Key Splashing Parameters of Remaining Slag

**DOI:** 10.3390/ma17235796

**Published:** 2024-11-26

**Authors:** Viktor Sinelnikov, Dorota Kalisz, Jan Novosád, Piotr Czarnywojtek, Cezary Rapiejko, Piotr Niedzielski, Rafał Kaczorowski, Pavel Srb, Breno Totti Maia, Michal Petrů, Katarzyna Ewa Łoś (Buczkowska)

**Affiliations:** 1 Cement Research Group, Łukasiewicz Research Network-Institute of Ceramics and Building Materials, Cementowa Str. 8, 31-983 Krakow, Poland; 2 Department of Chemistry and Corrosion of Metals, Faculty of Foundry Engineering, AGH University of Science and Technology, Al. A. Mickiewicza 30, 30-059 Krakow, Poland; dak@agh.edu.pl; 3 Department of Power Engineering Equipment, Faculty of Mechanical Engineering, Technical University of Liberec, Studentská Str. 2, 46117 Liberec, Czech Republic; jan.novosad@tul.cz; 4 Polytechnic Faculty, University of Kalisz, Nowy Świat Str. 4, 62-800 Kalisz, Poland; p.czarnywojtek@uniwersytetkaliski.edu.pl; 5 Department of Materials Technology and Production Systems, Faculty of Mechanical Engineering, Lodz University of Technology, Stefanowskiego Str. 1/15, 90-537 Lodz, Poland; cezary.rapiejko@p.lodz.pl (C.R.); rafal.kaczorowski@p.lodz.pl (R.K.); 6 Institute of Materials Science and Engineering, Faculty of Mechanical Engineering, Lodz University of Technology, Stefanowskiego Str. 1/15, 90-537 Lodz, Poland; piotr.niedzielski@p.lodz.pl; 7 Department of Machine Parts and Mechanism, Faculty of Mechanical Engineering, Technical University of Liberec, Studentská Str. 2, 46117 Liberec, Czech Republic; pavel.srb@tul.cz (P.S.); michal.petru@tul.cz (M.P.); 8 Lumar Metals, Rodovia MG 232, km 09, 70, Santana do Paraíso 35167-000, MG, Brazil; breno.totti@lumarmetals.com.br

**Keywords:** numerical modeling, recycling of waste material, supersonic jets, thermodynamic parameters of splashing, optimization of process

## Abstract

The paper is devoted to the analysis of a supersonic nozzle system effect in gas-cooled lances on the technological parameters of slag splashing in an oxygen converter. Simulation calculations were carried out, taking into account the parameters of nozzles used in the technological lines of converter steel plants in Ukraine and Brazil. The problems were solved in several stages. The simulation results of the first stage revealed the influence of different nozzle diameters *d_cr_*, *d_ex_* and the inlet pressure before nozzle *P*_0_ on the nitrogen consumption of one nozzle *V_н_*. Calculations also showed the influence of the critical *d_cr_* and output *d_ex_* of the nozzle diameter and nitrogen flow through one nozzle *V_н_* on the power of injected nitrogen *N*_1_ and the depth of penetration of the stream *h_x_* into the liquid slag. The second stage was dedicated to numerical simulation of the slag splashing process, including an array of results from the first stage. The thermodynamic and physical parameters were calculated using our own computer program, while 3D simulations were conducted using the ANSYS Fluent 2023 R2 program.

## 1. Introduction

Reducing wearing of the refractory lining in an oxygen converter by applying the slag splashing method has important value as, in this way, the cost of the smelting process can be reduced, the slag reused for repair purposes, and the loop economy implemented to some extent. Reducing the wearing away of the furnace lining is the target of research concentrating on refractory materials resistant to high temperatures and erosive agents. With the simultaneous use of a slag splashing method on the surface of worn-out lining elements, highly resistant furnace walls can be obtained, and the operating time significantly extended without necessary repairs [1,2,3].

Yang and co-authors [4] studied slag splashing in a 120 Mg converter using a lance equipped with a system of four de Laval nozzles at a Chinese steel mill. The authors analyzed the effect of the remaining slag in the converter and the influence of the oxygen lance position and nitrogen consumption at the top blow on the splashing slag. For this purpose, they used a *k*-*ε* turbulence model for isobaric jets [4,5]. The effects of different splashing parameters on the efficiency of the slag splashing process were analyzed by He et al. [5]. The authors studied the effect of nitrogen consumption on a profile of the impact crater formed in the slag during gas blowing, on the splash form, and on inclination using the Volume of Fluid method. During the experiment, the height of the lance position and gas consumption were adjusted through the bottom blow plug. In this way, it was revealed that uniform slag splashing could be achieved by adjusting the nitrogen consumption. Based on this, the authors [5] determined that the jet velocity for splashing should exceed 1.5 m/s. Another equally important finding was the optimal position of the lance over the liquid slag, thanks to which the jet velocity remained high, and the right contact surface with the slag was achieved for the best splashing effect.

On the other hand, studies by Maia et al. [6] were based on physical modeling, the results of which were compared with an industrial model. The authors presented the relationship between the parameters of the gas-cooled lance and their influence on the slag behavior. For this purpose, lances with a “yo-yo” effect were used. A traditional lance is held static during splashing, which leads the slag to flow down the walls of the converter and which is less efficient than in the “yo-yo” type, where the lance moves during the process. As a result, the stream of the sprayed slag had a greater dispersion and was not limited to one location; it covered several other locations simultaneously. Spraying was realized in different zones, in accordance with the defined lance position heights. Physical modeling was performed for two different slag levels and three system variants and nozzle angle variants, with one solution for a rotor nozzle, and the effect of the lance position height over the liquid slag was studied. The modeling was based on a comparavite characteristic of the parameters with the physical model, presented in the scientific work [7]. During the modeling of the physical object, polystyrene beads with a diameter of 3 mm were used. One of the most important aspects of the work was the use of a torsion nozzle, which introduces a new component, changing the overall behavior of the blown slag particles in a lower range.

The slag splashing studies previously carried out [7,8,9] helped us determine the optimal process parameters. It was observed that a high efficiency in the process can be achieved by optimizing a number of process parameters, e.g., flow parameters, pressure and temperature of the nitrogen stream, height and angle of the lance, the height of slag into which the gas stream enters, and MgO consumption. It should be noted that simulations were carried out for gas–powder streams, where MgO (m = 0–30 kg nitrogen/kg powder) was blown into the slag along with N_2_ to increase the lining adhesion. Numerical calculations in the program confirmed that the heating of the carrier (nitrogen) is beneficial to the slag splashing effect, as there is an additional temperature effect on the heating of the slag, which in turn affects its viscosity.

Sinelnikov et al. [7] studied the influence of the gas-cooled lance design on the slag splashing efficiency. The analysis of the calculation results revealed that cooling the lance with gas results in heat recovery, which increases the temperature of the injected mixture, so that the kinetic energy of the leaving stream in the converter increases by about 3.5 times, and the slag is splashed higher than in the case of a conventional water-cooled lance. In each of these works [8,9], only one de Laval nozzle system was considered. The analysis of the results of study [9] helped us effectively evaluate the gas-dynamic aspect of the splashing process. Researchers from the Pryazovskyi State Technical University related their research to the 350 mg converter of PJSC “Azovstal Iron & Steel Works” with top blowing. They considered the dissipation phenomenon of supersonic jet energy with the optimal selection of splash parameters. This problem was solved by changing the design of the lance, i.e., the water-cooled lance was replaced with a gas-cooled one, which in turn allowed them to increase the power and temperature of the jets before they entered the nozzle.

VOF (Volume of Fluid) modeling in ANSYS Fluent for industrial applications has been widely used. The most commonly used applications of the VOF model concern the free surface flow or movement of large bubbles in liquids [10]. There is tremendous strength in using this modeling approach, especially when studying microstructures where standard experimental techniques cannot be used. These are, e.g., microcapillaries [11] and similar small-scale structures. The team members’ previous VOF model application investigated the wettability of laser-structured surfaces [12].

The investigations in work [13] were focused on the development of a numerical 3D model including the VOF tool and the RNG *k*-*ε* turbulence model or LES model, to help understand the hydrodynamics in the slag stabilization processes. The team attempted to analyze the characteristic feature of gas injection into liquids with a very high viscosity index, commonly present in various processes related to chemical engineering. The flow characteristic of the TSL process was analyzed with a verified combination of models (i.e., the VOF-RNG/LES models) under industrially relevant conditions in industrial conditions.

A completely different concept in the theory of steelmaking processes and converter operations was proposed in [14,15]. The researchers proposed splashing the remaining slag with CO_2_ instead of the commonly used nitrogen to promote CO_2_ recycling in the steel industry. Splashing slag onto converter walls with CO_2_ streams is one of the technologies of the future, i.e., green metallurgy. For this purpose, a series of numerical simulations were performed comparing the characteristics of supersonic CO_2_, O_2_, and N_2_ streams. It was observed that when CO_2_ is used to splash slag, the oxygen lance must have an excellent splashing performance and mixing efficiency simultaneously. The process consists of four major steps [16]: waste gas purification, CO_2_ absorption, further CO_2_ gas regeneration, and slag splashing in supersonic CO_2_ jets. Some of the slag was left in the furnace after the steel was drained, and then powdered coke or coal was additionally fed into the furnace volume along with a slag modifier to ensure good fluidity of the slag. CO_2_ jets were then blown through an oxygen lance, causing the slag to splash onto the converter walls, forming a layer of high-melting slag to protect the furnace’s refractory lining. Importantly, during splashing, CO_2_ reacts with coke, generating twice as much CO, which not only improves mixing and splashing but also converts some of the CO_2_ into CO with a high heating value. When comparing the parameters of N_2_ and O_2_ with CO_2_, it was found that the CO_2_ stream has a lower velocity and dynamic pressure but a higher temperature at the outlet of the lance, and the difference between the three gases gradually decreases as the axial distance increases.

Slag splashing technology has been successfully used for over 30 years in converter steel plants. It is still being developed to improve the efficiency of the converter operation and extend the life of the refractory lining. Previous studies of the slag splashing process prove that there is no universal model for controlling the slag splashing process [4,5,6,7,8,9,10,11,12,13,14,15]. The slag splashing process cannot be universalized, which is due to the design of the converter (capacity: 40–400 Mg), the type of blowing (BOF converters with top blowing and with bottom blowing), the variety of grades of smelted steel, and the modes of operation of the converter related to the implementation of the oxygen- and gas-blowing process (blowing from above with a lance and from below through the bottom of the converter). For this reason, the operation of each converter has to be addressed.

The authors of this paper note that this investigation, presented in the current article, aimed to demonstrate to industrialists the thermodynamic phenomena during the use of de Laval supersonic nozzles, simplify the understanding and customization of gas/water cooling lances, and the most important aspect, assist metallurgists with choosing the optimal construction of lances based on the technological regime and thermodynamic aspects, such as the dissipation of transferred energy (nitrogen jets). These investigations were preceded by numerical [8,9] and physical simulations in a physical model of the oxygen converter [7], as well as pre-trials [6].

## 2. Materials and Methods

The first stage of this research was realized using a self-developed mathematical model and a private computer program for the “slag splashing” process. The is based on the compatibility of laboratory and industrial models [7,8,9]. It is important to note that this program was designed within Nationality Development Research Project “Development of technologies for the calculation of gas pipelines and pneumatic transport systems with a complex profile” at the Pryazovskyi State Technical University in Mariupol, Ukraine in 2013–2015. This program has passed a large number of studies with additional customization and industrial clarification, and ultimately, it has been implemented as a utility model for industrial processes within R&D and technology department of metallurgical plant in Ukraine. This thermodynamic program had been verified and validated accordingly. The process of verification included the following:

(1) A review (the process of reviewing documentation as well as changing it); b—design considering features and style; c—coding of the program;
(2) A walkthrough, including different starting points of investigations/data from the start;
(3) An inspectorate.

After that followed a validation process, which was based on the below stages:

(1) Unit tests (each of the modules used passed its own unit tests before the next step);
(2) Integrator tests (in this case, all modules were assembled);
(3) System testing involving customers’ specialist staff (this stage was carried out simultaneously on hardware and software, in customers’ departments);
(4) Admission comparative tests before and after the splashing process, including debugging activity (on the customer side, with the obligatory software developer presence and the institute’s thermodynamic and metallurgy specialist).

**Figure 1:**
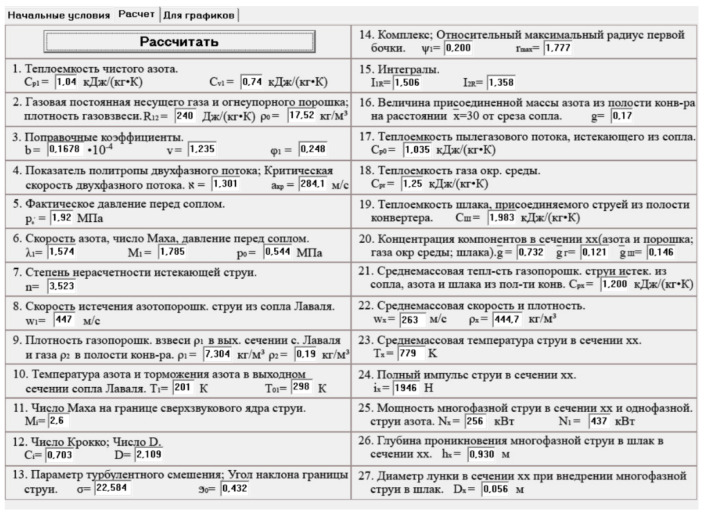
Window screen of the process parameter calculation program, which was created at the Pryazovskyi State Technical University in Mariupol, Ukraine (calculation example), where 1—heat capacity of pure nitrogen; 2—gas constant of the carrier gas and refractory powder; 3—correction coefficients; 4—polytropic index of a two-phase flow; 5—actual pressure before the nozzle; 6—velocity of nitrogen, Mach number, and pressure before the nozzle; 7—parameters of non-isobaric jet; 8—velocity of exhaust jet from the de Laval nozzle; 9—density of the gas–powder suspension at an outlet section of the de Laval nozzle and the gas in the cavity of the converter; 10—temperature of nitrogen and nitrogen deceleration in the outlet section of the de Laval nozzle; 11—Mach number at the boundary of the supersonic core of the jet; 12—Crocco number; 13—parameters of turbulent mixing, and the angle of inclination of the boundary jet; 14—relative maximum radius of the first cell in the mismatched supersonic jet; 15—integrals; 16—added mass of slag in the converter; 17—heat capacity of stream leaving the nozzle; 18—heat capacity of environment gas; 19—heat capacity of slag; 20—concentration of components in section *xx*; 21—mass average heat capacity of the exhausted jet; 22—mass average velocity and density; 23—stream temperature *T_x_* in section *xx*; 24—total impulse of the jet in section *xx*; 25—power of the multi-phase jet in section xx and the one-phased nitrogen jet; 26—jet penetration depth in slag in section *xx*; 27—diameter of hole in section *xx* during penetration of multi-phase jet in slag.

During the simulations, the turbulence parameters of the supersonic jet entering the slag were taken into account. Each simulation variant included a number of parameters of the slag splashing process. Figure 1 shows a calculation program window, while the data used from the simulation are listed in Table 1.

It is important to note that the physical aspects of supersonic jets that stream from de Laval nozzles into the cavity of oxygen converters were presented in previous studies [9,17,18]. However, developing the mathematical models of gas flow in supersonic nozzles requires paying attention to the peculiarity of the technological conditions of the splashing process. It is necessary to maintain a constant pressure in front of nozzles of the same size at the start of the spraying process. Nevertheless, this is technologically impossible, because oxygen supply machines produce different pressure levels at the time of starting splashing and at the time of finishing the process. The pressure drops from 1.7 MPa at the beginning of the slag splashing to 1.1 MPa at the finishing of the process. Furthermore, the flow stream remains in the supersonic speed range [9]. The nature of the supersonic flow that penetrates the volume of the converter is widely known and widely described in the literature. It is known that the nozzles of a nitrogen lance (as well as oxygen) can be designed and then manufactured for only one technological regime. This is calculated on the basis that the pressure at the nozzle section *P*_1_ and in the environment *P_g_* are equal, and the degree of non-isobaric n = *P*_1_/*P_g_*. However, it is never possible to withstand this technological regime.

There are significant opportunities to improve slag splashing technology, specifically by increasing the power of jets flowing from the lance and penetrating the converter’s volume and subsequently the slag alloy. It is often overlooked that during the interaction of high-energy jets with slag, the system’s entropy should not increase and the work of the supersonic jets should approach exergy. Furthermore, several of the previous studies [8,9,18] have shown that the efficiency of slag splashing can be significantly increased if the blowing gas (nitrogen), as well as the dispersed flow to the nozzles of the purge lance, are heated. Structurally, the lance should function as a heat exchanger, where the coolant—a gas mixture—absorbs heat from the high-temperature outgoing gas flows. This approach partially recovers heat that would otherwise be lost with the outgoing gases from the oxygen converter and creates conditions that enhance the efficiency (exergy) of the supersonic jet–slag system.

### 2.1. Mathematical Model of Thermodynamic Slag Splashing Program

The influence of the following parameters on the slag splashing process was analyzed: nitrogen temperature, nitrogen consumption, powder material consumption, and stream pressures for different variants. The applied mathematical model considers many parameters, the most important of which are as follows:

Heat capacity of nitrogen:

(1) $$c_{p_{1}}=\frac{k}{k-1}R_{1}$$

and

(2) $$c_{v_{1}}=\frac{1}{k-1}R_{1}$$

Constant *R*_12_ of carrier gas N_2_ and refractory powder (MgO) mixture:

(3) $$R_{12}=\frac{R_{1}}{1+\mu \psi }$$

where *µ* is the relation of mass charges of gas and powder coefficient = *m*_2_*/m*_1_; *m*_2_ is the mass consumption of nitrogen by a nozzle; *m*_1_ is the powder consumption; and ψ is the parameter of speed of flow = *w*_2_/*w*_1_.

The density of the gas stream (in the lance) is expressed with the following:

(4) $$\rho_{0}=\frac{p_{0}^{\prime }\left(1+\mu \right)}{R_{1}T_{0}}$$

The two-phase flow index is as follows:

(5) $$ℵ=\left(k-1\right)\left(1-\phi_{1}\right)+1$$

The critical value of two-phase flow equals the following:

(6) $$a_{kr}=\sqrt{\frac{2ℵ}{ℵ+1}\frac{1}{1+\mu \psi }R_{1}T_{0}}$$

The real pressure beyond the nozzle is described with the equation below:

(7) $$\rho_{0}^{\prime }=\frac{1}{B}\frac{m_{12}\sqrt{R_{12}T_{0}}}{F_{kr}}=\frac{1}{B}\frac{\rho_{n}V_{n}\left(1+\mu \psi \right)\sqrt{R_{12}T_{0}}}{F_{kr}}$$

where coefficient *B* is as follows:

(8) $$B=\sqrt{ℵ{\left(\frac{2}{ℵ+1}\right)}^{\frac{ℵ+1}{ℵ-1}}}$$

The density of the stream in the outlet nozzle cross-section is expressed with dependence:

(9) $$\rho_{1}=\rho_{0}\varepsilon \left(\lambda_{1}\right)=\frac{p_{0}^{\prime }\left(1+\mu \psi \right)\sqrt{R_{12}T_{0}}}{R_{1}T_{0}}{\left(1-\frac{ℵ-1}{ℵ+1}\lambda_{1}^{2}\right)}^{\frac{1}{ℵ-1}}$$

On the other hand, the nitrogen temperature on the outlet nozzle cross-section is expressed with the following:

(10) $$T_{1}=\frac{p_{1}}{\rho_{1}R_{1}}=\frac{np_{g}\left(1+\mu \psi \right)}{\rho_{1}R_{1}}$$

The temperature of nitrogen after it leaves the nozzle equals the following:

(11) $$T_{01}=T_{1}+\frac{w_{1}^{2}\left[ℵ-1\right]\left(1+\mu \psi \right)}{2ℵR_{1}}$$

where parameter $\psi_{1}$ takes the following form:

(12) $$\psi_{1}=\frac{1}{{\left(M_{i}^{2}-1\right)}^{1/2}}\frac{q\left(M_{i}\right)}{q\left(M_{1}\right)}$$

and parameter *I*:

(13) $$I_{1R}=\int_{-\infty }^{\eta_{R}}\frac{\phi d\eta }{\theta +\varphi \left(1-\theta \right)-\varphi^{2}C_{i}^{2}}$$

(14) $$I_{2R}=\int_{-\infty }^{\eta_{R}}\frac{\phi^{2}d\eta }{\theta +\varphi \left(1-\theta \right)-\varphi^{2}C_{i}^{2}}$$

The nitrogen stream in the volume of the converter at a distance x from the nozzle totals the following:

(15) $$g=\frac{2r_{max}}{D\sigma_{i}{\left(1-C_{i}^{2}\right)}^{1/2}\left(I_{1R}-I_{2R}\right)}$$

and thus, *C*—is the Crocco number:

(16) $$C=\sqrt{1-{\left(1-\frac{k-1}{2}M^{2}\right)}^{-1}}$$

where the following apply:

- *M*—Mach number at the boundary of the jet and the surrounding gas;
- *k*—ratio of specific heats;
- *D*—the coefficient, which establishes a relationship between the Mach number *M*_1_ at the nozzle cross-section and *n*:

(17) $$D={\left(\left(k-1\right)/2\right)}^{0.5}M_{1}n^{\frac{k+1}{2k}}$$

- *σ* = 12 + 2.58 *M*;
- *r_max_*—relative maximum radius of the first cell in the mismatched supersonic jet with density discontinuities, calculated by analogy [8,17,18];
- *x*—distance from the nozzle cross-section to the cross-section that is under consideration along the jet axis (measured in nozzle diameters), calculated with *l/r*_1_;
- *r*_1_—nozzle’s output radius.

The heat capacity of the stream leaving the nozzle is as follows:

(18) $$C_{p_{0}}=C_{p_{1}}{\bar{g}}_{1}+C_{2}{\bar{g}}_{2}$$

where the heat capacity of nitrogen:

(19) $$C_{p_{g}}=C_{p_{N_{2}}}=1.25$$

On the other hand, the heat capacity of slag equals the following:

(20) $$C_{sl}=0.276+1.138\cdot 10^{-3}t_{sl}$$

where *t_sl_* is the slag temperature.

The consumption of particular components in the process was described with the following expressions:

- Participation of nitrogen and MgO after leaving the nozzle:
  (21) $$\bar{g}=\frac{m_{1}}{m_{1}+m_{g}+m_{sl}}=\frac{1}{1+\frac{m_{g}}{m_{1}}+\frac{m_{sl}}{m_{1}}}=\frac{1}{1+g+g_{sl}}$$
- Participation of gas in the converter volume:
  (22) $${\bar{g}}_{g}=\frac{m_{g}}{m_{1}+m_{g}+m_{sl}}=\frac{1}{1+\frac{m_{1}}{m_{g}}+\frac{m_{sl}}{m_{g}}}=\frac{1}{1+\frac{1}{g}+\frac{g_{sl}}{g}}=\frac{1}{1+\frac{1}{g}\left(1+g_{sl}\right)}$$
- Participation of slag in the converter volume:
  (23) $${\bar{g}}_{sl}=\frac{m_{sl}}{m_{1}+m_{g}+m_{sl}}=\frac{1}{1+\frac{m_{1}}{m_{sl}}+\frac{m_{g}}{m_{sl}}}=\frac{1}{1+\frac{1}{g_{sl}}+\frac{g}{g_{sl}}}$$

The heat capacity of nitrogen and MgO after leaving the nozzle and slag in the converter volume was described with the relation below:

(24) $${c_{p}}_{x}=\sum c_{i}g_{i}={c_{p}}_{1}\bar{g}+{c_{p}}_{g}{\bar{g}}_{g}+c_{sl}{\bar{g}}_{sl}={c_{p}}_{1}{\bar{g}}_{1}+c_{2}{\bar{g}}_{2}+{c_{p}}_{g}{\bar{g}}_{g}+c_{sl}{\bar{g}}_{sl}\;={c_{p}}_{1}\frac{1}{1+\mu }+c_{2}\frac{\mu }{1+\mu }+{c_{p}}_{g}{\bar{g}}_{g}+c_{sl}{\bar{g}}_{sl}$$

The rate of N_2_ + MgO stream, nitrogen exiting the de Laval nozzle in the converter volume, and the associated slag stream is expressed with the following:

(25) $$w_{x}=\left[\left(1-g_{sl}\psi_{sl}\right)w_{1}+\frac{p_{g}\left(n-1\right)}{\rho_{1}w_{1}}-\frac{F_{x}}{F_{1}}\frac{p_{x}-p_{g}}{\rho_{1}w_{1}}\right]\frac{1}{\left(1+g+g_{sl}\right)\beta }$$

where the flow turbulence coefficient is about 1.03.

If *p_x_* = *p_1_*,

(26) $$w_{x}=\left[\left(1-g_{sl}\psi_{sl}\right)w_{1}+\frac{p_{g}\left(n-1\right)}{\rho_{1}w_{1}}\right]\frac{1}{\left(1+g+g_{sl}\right)\beta }$$

Stream temperature *T_x_* at a distance *x* from the nozzle cross-section is described with the relation below:

(27) $$T_{x}=\frac{T_{01}+g\frac{{c_{p}}_{g}}{{c_{p}}_{0}}T_{g}+g_{sl}\frac{c_{sl}}{{c_{p}}_{0}}T_{sl}-\alpha \left(1+g+g_{sl}\right)\frac{w_{x}^{2}}{2000}}{\left(1+g+g_{sl}\right)\frac{c_{p_{x}}}{c_{p_{0}}}}$$

The total stream pulse at a distance x from the nozzle cross-section equals the following:

(28) $$i_{x}=\left(m_{0}+m_{g}+m_{sl}\right)w_{x}=m_{1}\left(1+\mu \right)\left(1+g+g_{sl}\right)w_{x}=\rho_{n}V_{n}\left(1+\mu \right)\left(1+g+g_{sl}\right)w_{x}$$

The strength of the multiphase stream at a distance x from the nozzle cross-section is described with the formula below:

(29) $$N_{x}=\frac{\alpha \left(m_{1}+m_{g}+m_{sl}\right)w_{x}^{2}}{2000}=\frac{m_{1}\left(1+\mu \psi \right)\left(1+g+g_{sl}\right)w_{x}^{2}}{2000}$$

where α is the kinetic energy coefficient.

### 2.2. CFD Simulation of Slag Splashing

The second stage of this study involved simulations for computational fluid dynamics (CFD) calculations, which were carried out using the ANSYS Fluent 2023 R2 version [19,20]. CFD is a numerical method of simulating the behavior of systems, processes, and devices associated with gas and fluid flow, heat and mass transfer, chemical reactions, and other physical phenomena [21]. CFD programs help us obtain many important data on fluid flow (distribution of velocity field, pressure field), heat movement (temperature field), and other associated phenomena (including chemical reactions). This can be achieved by numerically solving equations that describe the movement of fluid mass and the energy balance.

**Figure 2:**
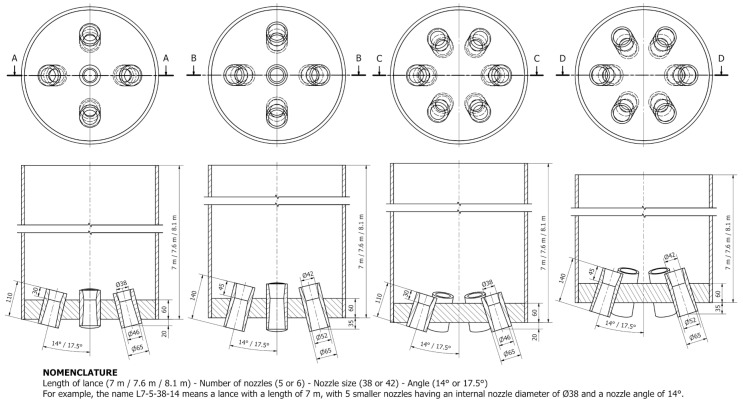
An arrangement of nozzles and their dimensions for simulations.

**Figure 3:**
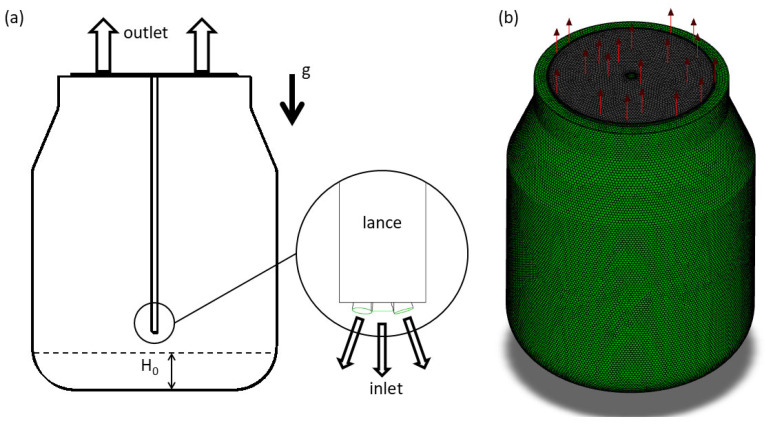
CFD simulation inputs: (**a**) geometry with the illustrated boundaries, (**b**) computation grid, g—the direction of gas (N_2_) injection, H_0_—initial slag height (i.e., the volume of remaining slag).

The geometry of the model is based on the oxygen converter [7]. In the axis of the converter, the lance ending with nozzles, illustrated in Figure 2, is placed. ANSYS SpaceClaim was then used for the preparation of the simplified fluid geometry for the CFD simulation. The 3D CAD model was discretized using the ANSYS Fluent Meshing tool to create the computational grid with polyhedral elements. The grid size was tuned to be applicable with the VOF model. Due to the scale of the converter, the smallest grid element was chosen to prevent the presence of discrete slag droplets smaller than 10 cm. The prismatic layers were created in the near-wall region with respect to the use of turbulence model wall functions. An illustration of the computational grid is shown in Figure 3b. The grid consists of approx. 120k cells for the half-symmetrical model.

In this study, the grid size was defined based on prior experience from similar cases [4,7], where the chosen mesh resolution demonstrated sufficient accuracy for comparable flow conditions. Although we have not carried out a detailed study of mesh independence, we have included the justification of the mesh size to be applied with the VOF model and references [12,13] based on our previous experience and the relevant literature to support our chosen mesh size.

A numerical solution was determined in ANSYS Fluent R2 version [19,20]. The solver was set as transient. The gravity model was applied with the direction of gravity acceleration from the top to the bottom of the convertor. A viscous *k-ε* model with the standard wall function approach was used. The multiphase VOF model was applied with the slag and gas as primary and secondary phases. Material properties followed the values in Table 2. Both fluids are considered incompressible with the constant density. Boundary conditions were applied as shown in Figure 3. Output sections of the nozzles were set as the inlet boundary, with the velocity value corresponding to the required nitrogen volumetric flow rate. The top convertor surface was defined as the pressure outlet to the surroundings. The rest of the boundary surfaces were labeled as walls, with a defined contact angle of slag at 120°. The second-order discretization schemes were applied. For the volume fraction equation, the Modified HRIC scheme was used.

To ensure that wall functions were appropriately applied, we monitored the *y*+ values throughout the domain. ANSYS Fluent’s automatic grid refinement feature was used to adaptively refine the mesh where necessary, particularly near walls, to maintain suitable *y*+ values for wall function use. The standard *k*-*ε* turbulence model was chosen due to its widespread application in similar studies and computational efficiency, especially given the large simulation domain [4,13].

The time of solution was set to 1 s. The initial computations showed that this time is enough to reach the developed flow regime inside the investigated domain. Adaptive time stepping was used with the initial time step at 10^−4^ s.

Hybrid initialization was performed before starting each calculation. Then, the region of slag defined by initial slag height H_0_ was patched with the volume fraction of slag equal to 1. The symmetry midplane was defined as a plane for results assessment. Velocity vector fields and the volume fraction of slag were evaluated during the calculation at various time steps. These data were saved each 0.01 s.

The 3D case CFD simulations were defined to study the influence of the following on the slag splashing process:

- Slag densities 2300 kg/m^3^ and 3000 kg/m^3^;
- Initial slag height analyzed for the range H_0_ = {0.5, 1.0, 1.5} m;
- Different numbers of nozzles: 1, 4, and 5 nozzles;
- Two nozzle outlet diameters: 46 mm and 52 mm;
- Two nozzle angles: 14° and 17.5°.

Due to the large number of calculation variants, different representative combinations of input parameters were selected in several stages.

## 3. Results and Discussion

### 3.1. Results of Thermodynamic Slag Splashing Simulation

**Figure 4:**
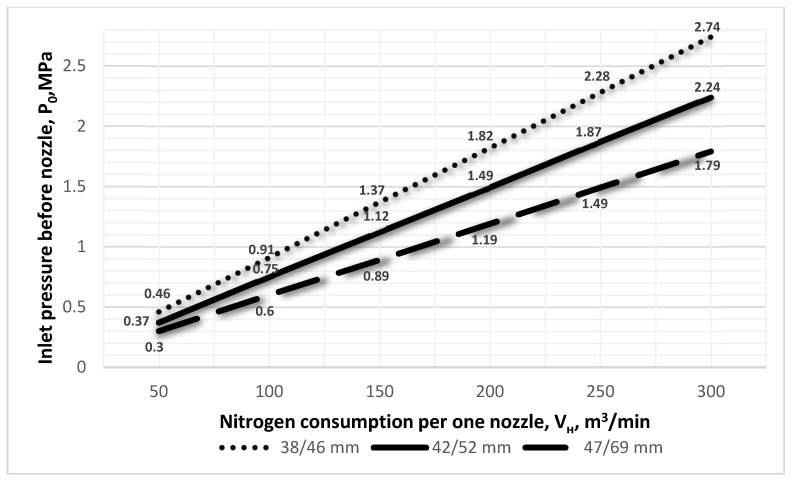
The effect of different de Laval nozzle diameters *d_cr_*/*d_ot_* and the nitrogen consumption per one nozzle *V_н_* on inlet pressure before nozzle *P*_0_.

Calculations with the authors’ computer private program were carried out for varying parameters of nozzle inlet and outlet diameters and varying nitrogen consumption levels per nozzle. The analysis of the results shown in Figure 4 reveals the effect of elevating the pressure before the nitrogen jet enters the supersonic nozzle at different final N_2_ flow rates through one nozzle *V_н_*, depending on the diameter of the de Laval nozzle. Attention should be paid to the very low difference in the pressure index *P*_0_ in front of the nozzle during splash regimes with low nitrogen consumption (Figure 4). This aspect is very important during the final stage of nitrogen blowing through the oxygen supply machine. As a consequence, the supersonic jet may have an inadequately low pulse, and consequently, insufficient power to lift the entire volume of the remaining slag. For example, when using a nozzle with a critical diameter of *d_cr_* = 47 mm and a diameter with an outlet cross-section of *d_ot_* = 69 mm and a nitrogen consumption of 50 m^3^/min by one nozzle, the pressure in front of the inlet cross-section of the nozzle was only 0.3 MPa, while with a five-fold increase in the amount of nitrogen flow *V_н_*, the pressure increased to a rate of 1.49 MPa.

**Figure 5:**
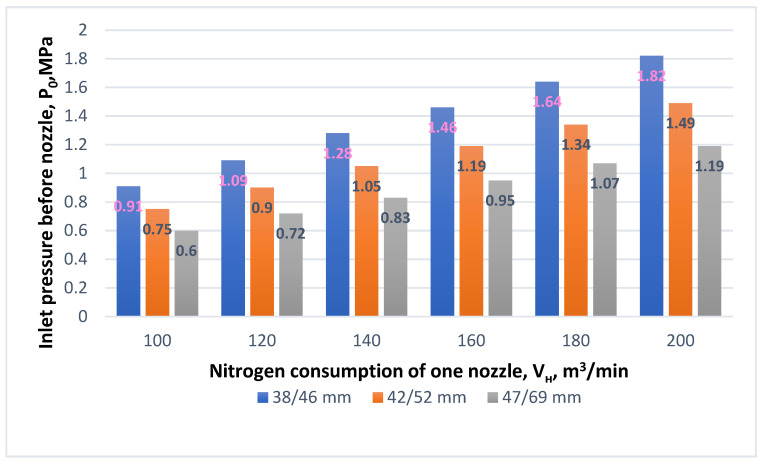
The effect of different de Laval nozzle diameters *d_cr_*/*d_ot_* and the nitrogen consumption of one nozzle *V_н_* on the inlet pressure before nozzle *P*_0_.

Next, the influence of de Laval’s supersonic nozzle diameter and nitrogen pressure before entering the nozzle on the final gas consumption per nozzle was analyzed. For *V_н_* = 140 m^3^/min and *d_ot_* = 47 mm, the pressure before the nozzle was 1.28 MPa, whereas for the same nitrogen consumption rate but with a larger exit nozzle diameter *d_ot_* = 52 mm, the pressure was 1.05 MPa. Simulation (Figure 5) showed that with the same nitrogen consumption rate and increased nozzle exit diameter, the inlet pressure in front of the nozzle decreased.

**Figure 6:**
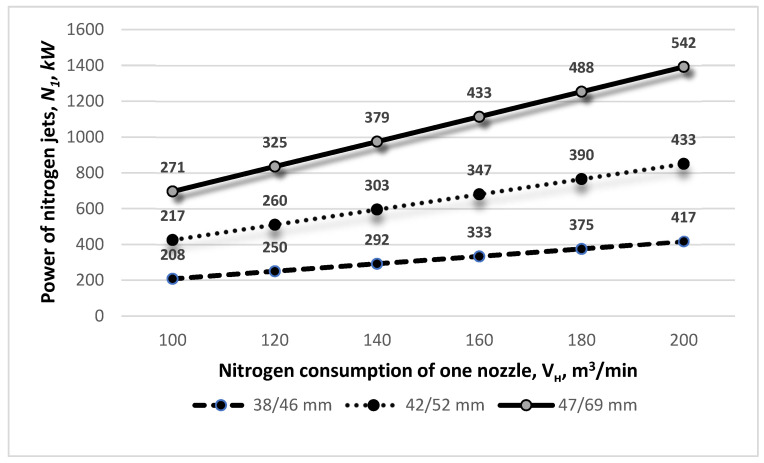
The influence of different de Laval nozzle diameters *d_cr_*/*d_ot_* and the nitrogen consumption of one nozzle *V_н_* on the power of nitrogen jets *N*_1_.

Next, the effect of changing the N_2_ consumption of a single nozzle on the power of the supersonic nitrogen jet was analyzed. It was observed that with the increasing nitrogen consumption of a single nozzle *V_н_*, the power of the nitrogen jets increased. As can be seen from Figure 6, for a critical nozzle diameter of 38 mm and an outlet diameter of 46 mm, and a nitrogen consumption per nozzle of 120 m^3^/min, the nitrogen jet power N_1_ is 0.250 MW. However, for the same nozzle size, with the nitrogen consumption elevated to 200 m^3^/min, the power of the supersonic N_1_ gas jet increases to a rate of 0.417 MW.

**Figure 7:**
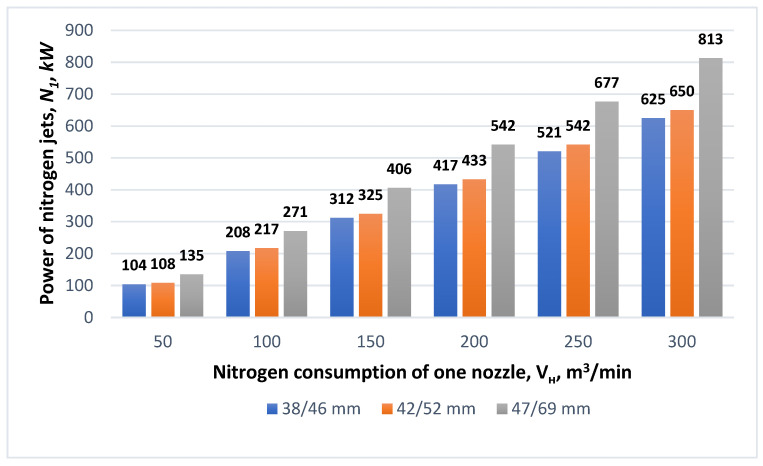
The influence of different de Laval nozzle diameters *d_cr_*/*d_ot_* and the nitrogen consumption of one nozzle *V_н_* on the power of nitrogen jets *N*_1_.

It should be noted, however, that the operation of large-volume oxygen converters in the slag splashing process in the high-nitrogen-consumption modes clearly affects the supersonic jet power. As can be seen in Figure 7, when using supersonic nozzles with diameters *d_cr_, d_ot_* = 47 mm and 69 mm, respectively, and nitrogen consumption *V_н_* = 50 m^3^/min, the power of the N_1_ jet equals 0.135 MW; when the nitrogen flow rate *V_н_* is increased to 300 m^3^/min, the power of the supersonic nitrogen jet is as high as 0.813 MW.

**Figure 8:**
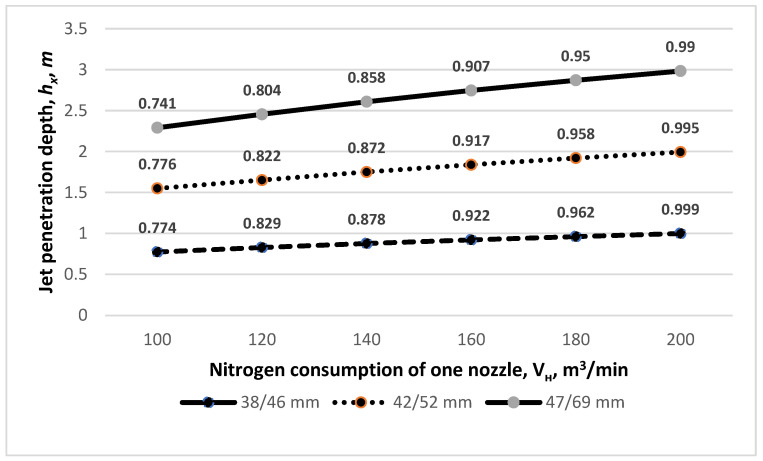
The influence of different de Laval nozzle diameters *d_cr_*/*d_ot_* and the nitrogen consumption of one nozzle *V_н_* on the jet penetration depth *h_x_*.

**Figure 9:**
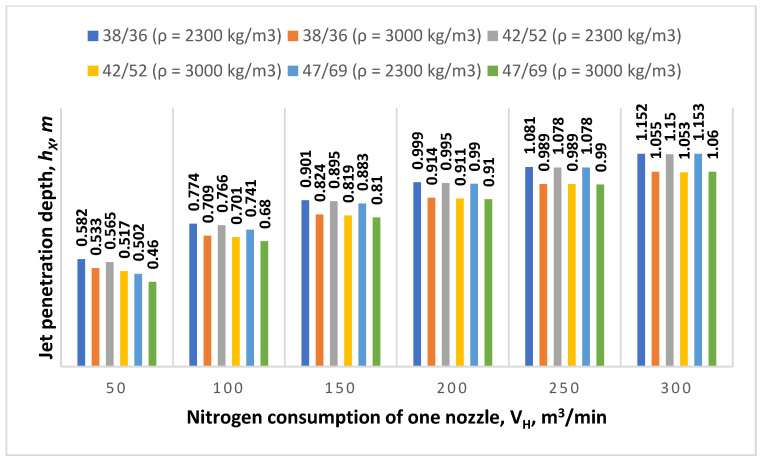
The influence of different de Laval nozzle diameters *d_cr_*/*d_ot_* and the nitrogen consumption of one nozzle *V_н_* on the jet penetration depth *h_x_*.

Next the authors studied the influence of gas flow through the de Laval nozzle on the amount of immersion of this jet in the liquid slag. This relationship is very important as on this basis, one can predict the behavior of the jet and the necessary pulse for splashing depending on the volume of slag remaining after smelting, which in turn significantly helps control the converter furnace during the slag splashing process. The analysis of the obtained results reveals that with the increasing nitrogen consumption *V_н_*, the depth of immersion of the supersonic jet in the slag increases, too. When using nozzles with *d_cr_, d_ot_* = 38 mm and 46 mm diameters and a nitrogen flow rate of 100 m^3^/min, the depth of penetration is 0.774 m, while with the same diameters and *V_н_*= 180 m^3^/min, the penetration increases to 0.962 m (Figure 8). On the other hand, when using supersonic nozzles with diameters of *d_cr_, d_ot_* = 47 mm and 69 mm and the same nitrogen consumption rates, the penetration depths decrease to 0.741 and 0.95 m. The depth of penetration into the slag stream depends on its physicochemical parameters. A decrease in the depth of penetration of the supersonic jet into the slag melt is observed with an increase in slag density. For example, for a slag density of 2300 kg/m^3^ and the operation of a lance with supersonic nozzles with diameters of *d_cr_, d_ot_* = 42 mm and 52 mm, respectively, and a nitrogen consumption per nozzle of 200 m^3^/min, the depth of penetration is 0.995 m, while for the same nozzle rates and a slag density increased to 3000 kg/m^3^, the depth of the jet’s immersion in the slag decreases to 0.911 m (Figure 9).

### 3.2. Results of CFD Simulation

**Figure 10:**
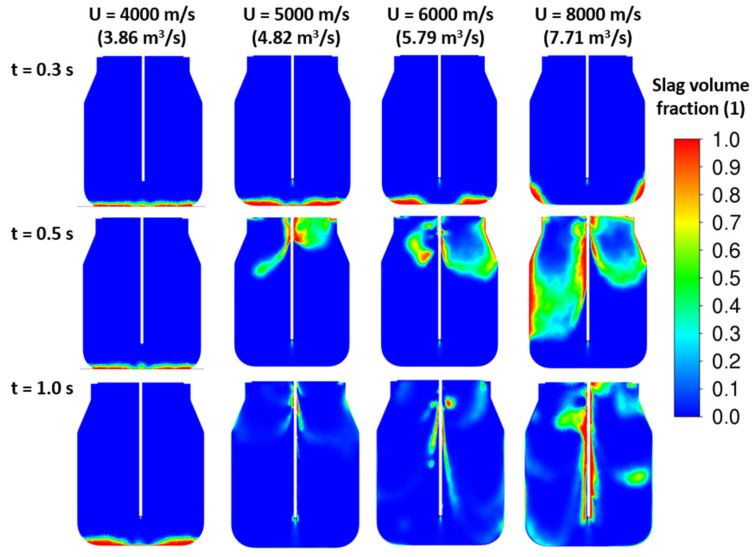
CFD results: influence of the inlet velocity—1 nozzle, ρ*_sl_* = 3000 kg/m^3^, H_0_ = 0.5 m.

The Fluent solver settings were described in Section 2 in this article. The initial computation stage started with one central nozzle at different inlet velocities corresponding to different nitrogen volumetric flow rates. The slag volume fraction contours represent the distribution of slag inside the convertor, where the value represents the dimensionless ratio of the slag volume to the total volume of each grid cell. The slag volume fraction value equal to 0 means only the presence of gas with no slag, and the value equal to 1 means only the presence of slag in the cell volume. The results for time development with the slag density 3000 kg/m^3^ are shown in Figure 10. The velocity values are set to be consistent with the nitrogen flow rates. Due to the incompressibility assumption, the constant density causes an overprediction of the velocity values. Based on the solved cases, the one-nozzle configuration seems not to be sufficient for slag splashing. The increase in velocity only decreases the time to reach the full penetration of the initial slag layer. The impact of a high-velocity jet on the bottom wall creates slag movement to the sides. To conclude, the variant with one central nozzle causes the movement of slag to the sides but not its splashing and recirculation.

**Figure 11:**
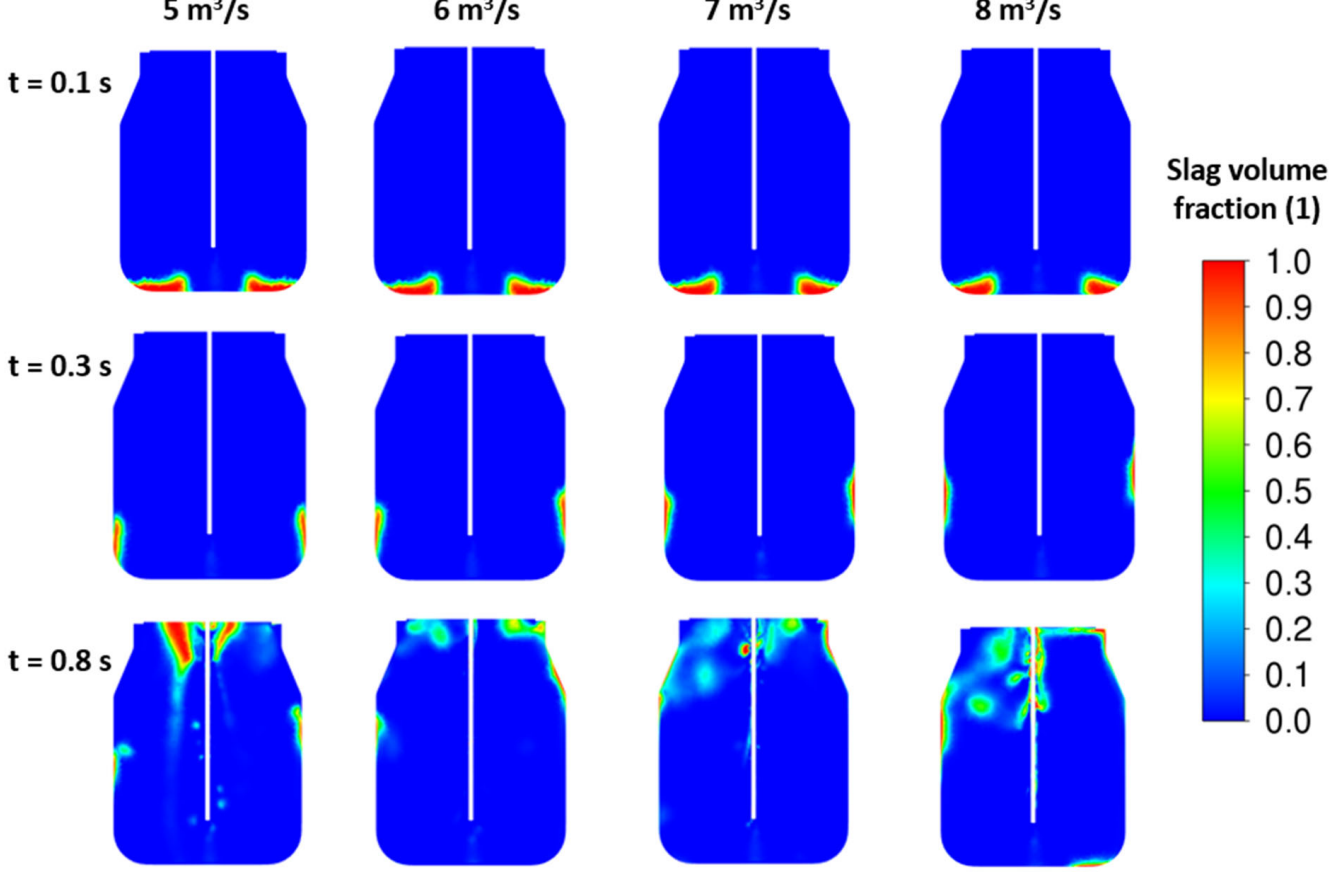
CFD results: influence of the inlet velocity—4 nozzles, ρ*_sl_* = 3000 kg/m^3^, H_0_ = 0.5 m.

**Figure 12:**
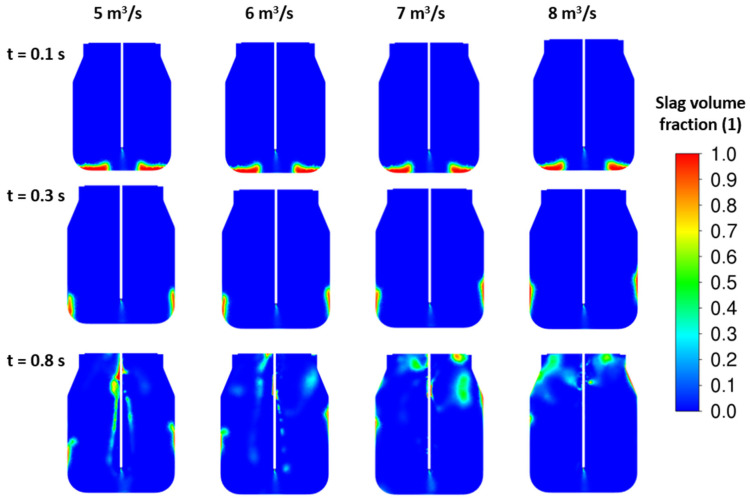
CFD results: influence of the inlet velocity—5 nozzles, ρ*_sl_* = 3000 kg/m^3^, H_0_ = 0.5 m.

To improve the splashing process, more than one nozzle should be applied. New sets of results are illustrated in Figure 11 and Figure 12 for four- and five-nozzle cases. Based on the slag fraction contours, the four-nozzle case seems to be more intense in the impact of the jet. Here, the higher nozzle outlet velocity, i.e., higher jet kinetic energy, influences the height of the peak after the first lift off of the initial slag layer. The amount of slag corresponding to the initial height H_0_ = 0.5 m is in full motion for all cases. Finally, the total energy of the slag layer is lower than the total jet energy, which causes the slag to blow out of the converter.

**Figure 13:**
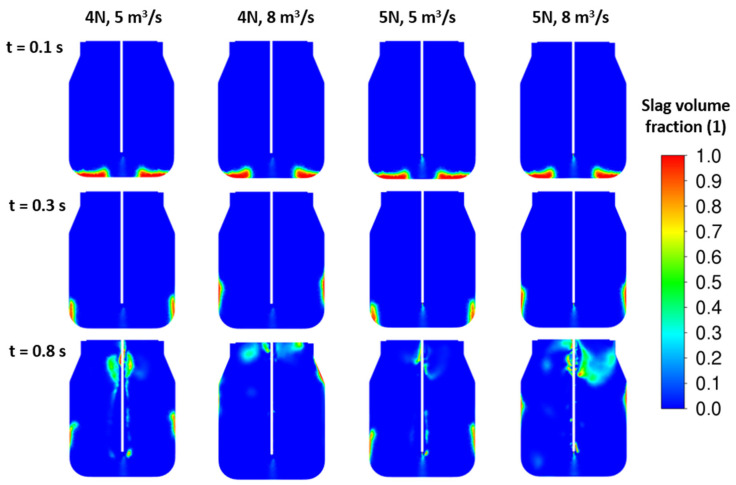
CFD results: d = 46 mm, α = 14°, ρ*_sl_* = 2300 kg/m^3^, H_0_ = 0.5 m.

**Figure 14:**
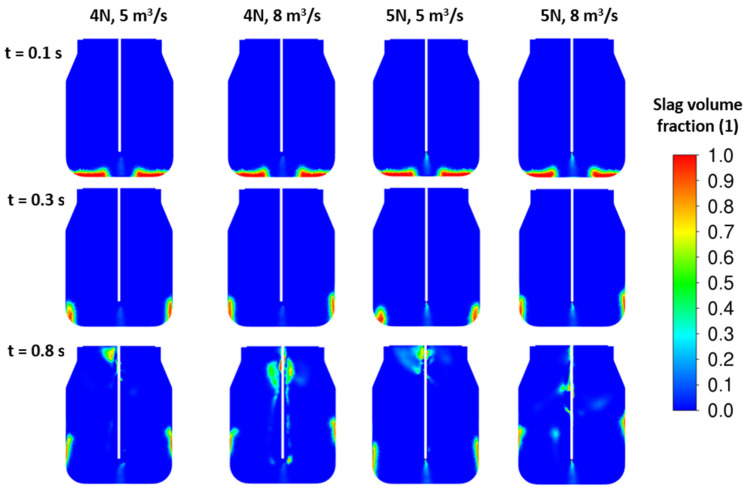
CFD results: d = 52 mm, α = 14°, ρ*_sl_* = 2300 kg/m^3^, H_0_ = 0.5 m.

The change in slag density shown in Figure 13 does not significantly influence the results for any investigated nozzle version. Contours in Figure 14 concern the increased nozzle diameter. Due to the decrease in velocity, the presented results lead to a worse situation than the previous cases. The results of the tested variants show a dominant effect of velocity on slag splashing, where the combination of the velocity magnitude, its spatial distribution through multiple nozzles, and the total energy required to set the slag in motion play a role.

**Figure 15:**
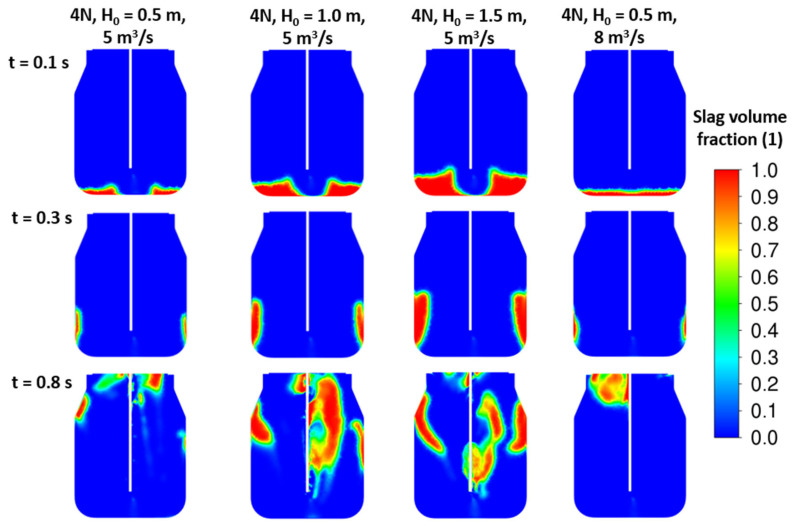
CFD results: 4 nozzles, d = 46 mm, α = 17.5°, ρ*_sl_* = 2300 kg/m^3^, various initial slag heights.

**Figure 16:**
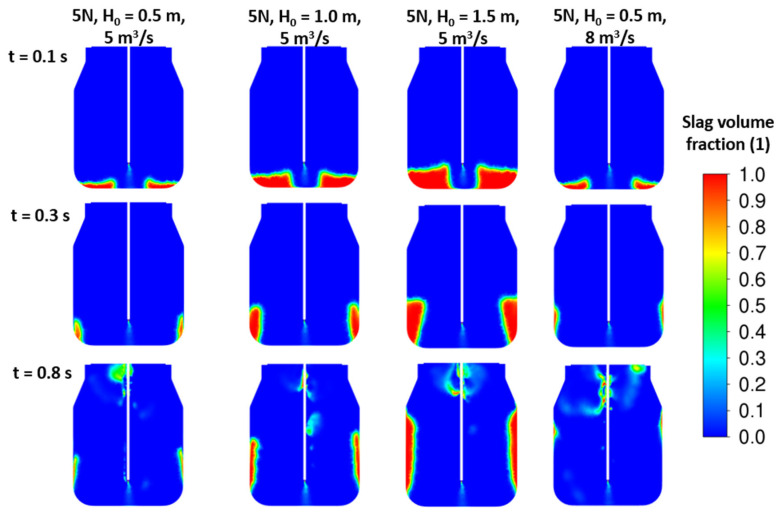
CFD results: 5 nozzles, d = 46 mm, α = 17.5°, ρ*_sl_* = 2300 kg/m^3^, various initial slag heights.

**Figure 17:**
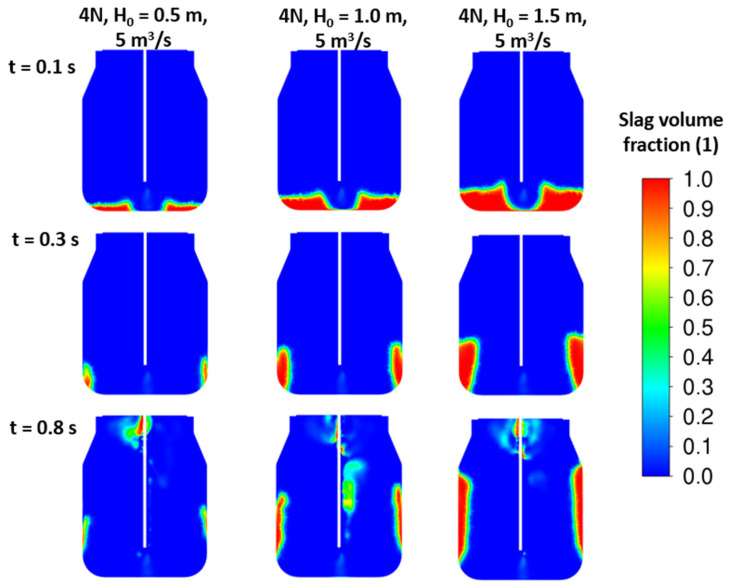
CFD results: 4 nozzles, d = 52 mm, α = 17.5°, ρ*_sl_* = 2300 kg/m^3^, various initial slag heights.

The initial slag height variations were studied to gain a clear understanding of the energy balance of the jet and slag mass. Three initial height cases of H_0_ = (0.5; 1.0; 1.5) meters were studied for different nozzle dimensions and arrangements. As shown in the results in Figure 15, Figure 16 and Figure 17, especially at time 0.3 s, there is a significant deflection of the slag away from the converter wall, which seems to have a positive effect on its circulation and spraying in the lower part of the converter.

Finally, it should be emphasized that the calculations were carried out in such a way that the slag was not splashed into droplets smaller than approximately 10 cm. This dimension was determined by the cell size of the computational grid used. Based on the presented results, it is, therefore, possible to evaluate the behavior of different nozzle configurations and operating conditions. However, the computational methodology used does not allow us to conclusively predict the behavior of the slag after it has been agitated, especially in the area of recirculation and drift, where the formation of small droplets and their further distribution in the converter space will also play a significant role.

## 4. Conclusions

1. The effect of the different dimensions of supersonic nozzles of converter slag on the thermodynamic and technological parameters was analyzed from the perspective of applying slag splashing technology.
2. Simulation with our own thermodynamic program, Slag Splashing, allowed us to determine of influence the de Laval nozzle diameters and nitrogen consumption per one nozzle *V_н_* on the power of nitrogen jets *N*_1_, inlet pressure before nozzle *P*_0_, and jet penetration depth *h_x_*.
3. The simulation in ANSYS Fluent (CFD method) showed that splashing with one nozzle is inefficient, in the case of using a velocity of 4000 m/s and nitrogen consumption per one nozzle of 3.86 m^3^/s, and that this impulse is not enough for lifting, even with a large quantity of slag, H_0_ = 0.5 m. However, when increasing the nitrogen consumption of one nozzle to 4.82 m^3^/s, 5.79 m^3^/s, or 7.71 m^3^/s, we can observe that the impulse of the supersonic jet is sufficient to lift the total amount of slag, but this causes splashing non-uniformly along the entire height of refractory lining.
4. Comparative simulations were carried out for lances with four and five nozzles using different densities of slag and various angles of nozzle inclination. The simulation also included variations in nitrogen flow per nozzle and various initial slag heights. The observed results show that the most universal and uniform variant is that with five nozzles and a nitrogen consumption per nozzle of 5 m^3^/s, allowing the largest amount of slag to spread on the surface of the refractory lining. We should also note the variant with four nozzles and a nitrogen consumption per one nozzle of 5 m^3^/s, with a height of the lance above the slag of 1.5 m. This ratio of technological parameters (balance) allows us to get a more controlled slag flow.
5. The results obtained in this article can be used by industrialists during the formulation of recommendations for the industrial process, as well as before the process, for predicting the rate of wear (durability) of refractory linings.

## Figures and Tables

**Table 1:** Selected source data for mathematical simulation [6,7,8,9].

No	Simulated Parameters	Index
1	Temperature of gas and powder mixture	t*_o_* = 25 °C
2	Density of molten converter’s slag	ρ*_sl_* = 2300, 3000 kg/m^3^
3	Temperature of gases in converter	t_g_ = 1500 °C
4	Pressure of gases in converter	p_g_ = 0.1 MPa
5	Heat capacity of nitrogen	$c_{p_{1}}$ = 1.25 KJ/(kg·K)
6	Nitrogen consumption by one nozzle	V*_н_* = 50–300 m^3^/min
7	Nozzle diameter at a critical place	d*_cr_*= 38–47 mm
8	Nozzle diameter at an outlet	d*_ot_*= 46–69 mm

**Table 2:** Experimental parameters for first geometry—inputs for simulation.

Quantity of Nozzles	Density of Gas, kg/m^3^	Viscosity of Gas, Pa·s	Density of Slag, kg/m^3^	Viscosity of Slag, Pa·s	Nitrogen Flow Through One Nozzle, m^3^·s^−1^	Initial Slag Height, m	Height of the Lance Above Slag, m	Surface Tension of Liquid Slag, N·m^−1^
1	1.225	0.00006	3000	0.07	8	0.5	1.5	0.5
4	1.225	0.00006	3000	0.07	2.0	0.5	1.5	0.5
5	1.225	0.00006	3000	0.07	1.6	0.5	1.5	0.5

## Data Availability

The original contributions presented in the study are included in the article, further inquiries can be directed to the corresponding authors.
